# A novel *de novo QRICH1* variant causing Ververi–Brady syndrome with infantile epileptic spasms syndrome: clinical and genetic analysis

**DOI:** 10.3389/fnins.2026.1848591

**Published:** 2026-06-15

**Authors:** Hongjun Fang, Xinghan Wu, Xiaojun Kuang, Liwen Wu, Xiao Zhang, Lijuan Wang, Sai Yang, Hongmei Liao, Zeshu Ning

**Affiliations:** 1 Department of Neurology, The Affiliated Children's Hospital of Xiangya School of Medicine, Central South University (Hunan Children's Hospital), Changsha, China; 2 Department of Medical Genetics, The Affiliated Children's Hospital of Xiangya School of Medicine, Central South University (Hunan Children's Hospital), Changsha, China

**Keywords:** clinical phenotype, epilepsy, gene mutation, infantile epileptic spasms syndrome, *QRICH1* gene, Ververi–Brady syndrome

## Abstract

**Objective:**

This study aims to investigate the clinical phenotype and genetic etiology of a case of Ververi–Brady syndrome (VBS) with infantile epileptic spasms syndrome (IESS) caused by a novel *de novo* variant in the *QRICH1* gene.

**Methods:**

Clinical data were retrospectively collected from a pediatric patient admitted to Hunan Children’s Hospital on July 28, 2025, due to intermittent nodding episodes for 10 days. Trio-based whole-exome sequencing (trio-WES) was performed for the proband and his parents. Candidate variants were validated by Sanger sequencing and assessed for pathogenicity. Relevant literature was reviewed to summarize genotype–phenotype correlations.

**Results:**

The patient, a 5-month-and-22-day-old male infant, presented with facial dysmorphism, global developmental delay, and IESS. After treatment with adrenocorticotropic hormone (ACTH) and vigabatrin, seizures were fully controlled and developmental outcomes improved. Trio-WES identified a novel heterozygous frameshift variant in the *QRICH1* gene (NM_198880.3: c.1282dup, p. Gln428Profs*27), which was *de novo* and absent in both parents. According to the ACMG/AMP guidelines, this variant was classified as pathogenic (PVS1 + PS2 + PM2_Supporting). A literature review identified 11 relevant articles, encompassing a total of 46 patients (including the present case) with 41 distinct *QRICH1* variants: 10 missense, 11 nonsense, 17 frameshift, and 3 splicing mutations. Common clinical features included developmental delay, nonspecific facial dysmorphism, hypotonia, autism spectrum disorder, epilepsy, and scoliosis.

**Conclusion:**

*QRICH1* variants underlie Ververi–Brady syndrome. Here we describe a patient with *QRICH1*-related Ververi–Brady syndrome presenting with IESS. Combined treatment with ACTH and vigabatrin was followed by seizure freedom, electroencephalographic (EEG) improvement, and developmental gains in this patient. This report expands the genotypic and phenotypic spectrum of the disorder.

## 1 Introduction

Ververi–Brady syndrome (VBS) is a rare hereditary neurodevelopmental disorder characterized by developmental delay, intellectual disability, mild facial dysmorphism, hypotonia, short stature, and variable epilepsy. Since its first report in 2018, fewer than 50 cases have been documented worldwide. *QRICH1* (Q-rich protein 1) is the established causative gene for this disorder; however, its precise pathogenic mechanism and genotype–phenotype correlation require further investigation (Kumble et al., 2022; Ververi et al., 2018). The protein encoded by *QRICH1* plays a critical role in the endoplasmic reticulum stress response and maintains cellular function by regulating proteostasis (You et al., 2021). Dysfunction of *QRICH1* impairs its key function in governing selective mRNA translation under cellular stress, leading to dysregulated synthesis of proteins essential for neuronal development and synaptic function, and ultimately resulting in neurodevelopmental impairment (You et al., 2021; Wang et al., 2025; Martínez et al., 2018; Mignogna et al., 2015). This study presents the clinical and genetic analysis of a pediatric patient diagnosed with VBS caused by a heterozygous *QRICH1* variant at Hunan Children’s Hospital. The patient presented with distinctive facial dysmorphism, global developmental delay, and infantile epileptic spasm syndrome (IESS). We report a case of VBS with IESS caused by a *QRICH1* gene variant, which not only expands the genotypic and phenotypic spectrum of this disorder but also provides new evidence for its clinical diagnosis and management.

## 2 Subjects and methods

### 2.1 Subjects

A male patient with Ververi–Brady syndrome who was admitted to Hunan Children’s Hospital on July 28, 2025, due to intermittent nodding attacks for 10 days was enrolled in this study. The study was approved by the Medical Ethics Committee of Hunan Children’s Hospital (approval number: HCHLL-2025-397). Written informed consent was obtained from the patient’s guardians.

### 2.2 Methods

#### 2.2.1 Clinical data collection

Clinical data were collected, including general information (gender, age, disease course), medical history (present illness, past history, personal history, family history), physical examination, auxiliary examinations, treatment, and follow-up data.

#### 2.2.2 Whole-exome sequencing and bioinformatics analysis

Trio-WES was performed for the proband and his parents. Peripheral venous blood samples (3 mL) were collected from the patient and his parents, respectively, and anticoagulated with EDTA. Genomic DNA was extracted using the BloodGen Midi Kit (Tiangen Biotech Co., Ltd., Beijing, China). Library preparation was performed by DNA fragmentation, capture, and enrichment. Exonic regions were captured using the xGen® Exome Research Panel v2.0 (IDT, USA), and sequencing was performed on the Illumina HiSeq X Ten platform with 150-bp paired-end reads.

Bioinformatics analysis was carried out using the ISoGenetic analysis system integrated with Sentieon software (202308.03) for accelerated data processing. Raw sequencing data were aligned to the human reference genome hg19 (GRCh37) using BWA-MEM (v0.7.19). The aligned BAM files were sorted, deduplicated, and indexed using Sentieon, Samtools (v1.15.1), and Picard (v1.119) to generate clean BAM files and corresponding BAI index files. Data quality control and target region coverage evaluation were performed using bamdst (v1.0.9) and QualiMap (v2.2.1). Variant calling for single nucleotide variants (SNVs) and small insertions/deletions (indels) strictly followed the GATK Best Practices workflow as implemented in Sentieon. Variant annotation was performed using ANNOVAR with databases including gnomAD v2.1.2, ClinVar, and HGMD. Parent–child genotype consistency in trio-WES confirmed maternity and paternity. Pathogenicity of variants was classified according to the 2015 guidelines of the American College of Medical Genetics and Genomics (ACMG) and the Association for Molecular Pathology (AMP) (Richards et al., 2015).

#### 2.2.3 Sanger sequencing validation

Candidate variants identified by whole-exome sequencing and data analysis were validated by Sanger sequencing. Primers were designed using the online software NCBI Primer-BLAST. PCR products were purified using a Tiangen purification kit and sequenced on an ABI-3500DX sequencer (Applied Biosystems, USA). Data were analyzed using SeqMan software. The primer sequences were as follows: *QRICH1*_E4Fseq: CAGCAGCAGAGCATTACCCA; *QRICH1*_E4R: AATAAGCCAGAGGTTGCGAG.

#### 2.2.4 Literature review

To systematically review the research progress of Ververi–Brady syndrome and the *QRICH1* gene, relevant literature was searched in the Wanfang Data Knowledge Service Platform, PubMed, HGMD, and ClinVar databases using the keywords: Ververi–Brady syndrome, *QRICH1* gene variation, epilepsy. The search period was from the inception of each database to December 29, 2025. Studies were included if they reported original clinical and genetic data of patients with *QRICH1* variants; reviews and articles without detailed individual patient data were excluded. After screening, studies focusing on *QRICH1* variant types and clinical phenotypes were analyzed. Phenotype frequencies were calculated for each clinical feature separately. For each phenotype, only patients with available data (marked “+” or “−”) were included in the denominator; patients with missing data for that feature (marked “/” in Table 1) were excluded from the calculation to avoid bias.

**Table 1:** Summary of clinical and genetic characteristics in 46 patients with *QRICH1*-related Ververi–Brady syndrome.

Pt	Ref.	Sex	Age	Variant	Variant type	ID	Facial dysmorphism	Hypotonia	Poor weight gain	Short stature	Autism	Seizures (age of onset)	Scoliosis	Brain MRI abnormalities
1	Kumble et al. (2022)	M	10y	c.46C > T, p.(Arg16*)	Nonsense	Normal	+	−	−	−	+	+, 8y	−	−
2	Kumble et al. (2022)	M	11y	c.136del, p.(Gln46Serfs*200)	Frameshift	Mild	+	−	−	−	+	−	−	+
3	Ververi et al. (2018)	F	9y	c.138_139delinsTT, p.(Gln46_Gln47delinsHis*)	Nonsense	Mild	+	−	+	+	−	−	−	−
4	Baruch et al. (2021)	M	4y	c.246del, p.(Ser83Valfs*163)	Frameshift	Mild	+	−	+	−	−	−	−	−
5	Kumble et al. (2022	M	22m	c.310-2A > C, p.(?)	Splicing	Normal	+	+	+	+	−	−	−	−
6	Kumble et al. (2022)	F	10y	c.541C > T, p.(Gln181*)	Nonsense	Normal	+	+	−	−	−	−	+	−
7	Kumble et al. (2022)	F	3y	c.756G > T, p.(Met252Ile)	Missense	Severe	+	−	−	−	−	−	−	+
8	Kumble et al. (2022)	F	13y	c.823C > T, p.(Gln275*)	Nonsense	Mild	+	+	−	−	−	−	+	−
9	Föhrenbach et al. (2021)	F	15.25y	c.832_833del, p.(Ser278Leufs*25)	Frameshift	Normal	+	−	−	−	−	−	+	+
10	Kumble et al. (2022)	M	11y	c.851C > T, p.(Pro284Leu)	Missense	Moderate	+	−	−	−	+	+, 5y	−	−
11	Kumble et al. (2022)	M	26y	c.914dup, p.(Gly306Argfs*48)	Frameshift	Mild	+	−	+	−	+	−	−	−
12	Kumble et al. (2022)	F	13y	c.961del, p.(Asp321Thrfs*47)	Frameshift	Mild–Moderate	+	−	+	−	−	+, 6y	−	−
13	Kumble et al. (2022)	M	13y	c.985del, p.(His329Thrfs*39)	Frameshift	Moderate–Severe	+	−	−	+	−	−	−	+
14	Kumble et al. (2022)	M	7y	c.1147_1150del, p.(Leu383Phefs*6)	Frameshift	Mild	+	−	−	−	+	−	−	−
15	Kumble et al. (2022)	M	15y	c.1180C > G, p.(His394Asp)	Missense	Mild	+	+	−	−	+	+, 14y	−	−
16	Kumble et al. (2022)	F	11y	c.1258C > T, p.(Gln420*)	Nonsense	Severe	+	+	+	+	−	+, 6 m	+	+
17	Kumble et al. (2022)	F	14y	c.1292dup, p.(Pro432Thrfs*23)	Frameshift	Normal	+	−	+	+	−	−	+	−
18	Kumble et al. (2022)	F	6y	c.1304A > G, p.(Gln435Arg)	Missense	Normal	+	−	+	−	−	−	−	−
19	Cope et al. (2020)	F	15y	c.1378C > T, p.(Gln460*)	Nonsense	Mild	+	−	−	−	−	+, 6y	−	+
20	Lui et al. (2019)	M	11y	c.1531C > T, p.(Arg511*)	Nonsense	Mild	+	−	−	−	−	−	−	−
21	Kumble et al. (2022)	M	6y	c.1579G > A, p.(Gly527Arg)	Missense	Mild	+	+	+	+	−	−	−	−
22	Lui et al., 2019)	F	8y	c.1606C > T, p.(Arg536*)	Nonsense	/	+	+	+	+	−	−	+	−
23	Kumble et al. (2022)	M	15y	c.1626del, p.(Tyr543Metfs*23)	Frameshift	Moderate	+	−	−	−	−	−	−	+
24	Kumble et al. (2022)	F	6y	c.1649A > G, p.(Tyr550Cys)	Missense	Normal	+	+	−	+	−	−	−	−
25	Kumble et al. (2022)	M	8y	c.1655del, p.(Phe552Serfs*14)	Frameshift	Moderate	+	−	−	+	−	−	−	−
26	Kumble et al. (2022)	M	14y	c.1720 T > G, p.(Tyr574Asp)	Missense	Normal	−	−	−	−	+	+, 9y	−	−
27	Kumble et al. (2022)	M	3y	c.1787-2A > G, p.(?)	Splicing	/	+	−	−	+	−	−	−	−
28	Wang and Wu (2023)	F	5y	c.1788dupC, p.(Tyr597Leufs*9)	Frameshift	Mild	+	−	+	+	−	−	−	−
29	Kumble et al. (2022)	M	3y	c.1807G > T, p.(Val603Leu)	Missense	Normal	−	+	−	−	+	+	−	−
30	Föhrenbach et al. (2021)	M	12.5y	c.1812_1813del, p.(Glu605Glyfs*25)	Frameshift	Normal	+	−	+	−	−	−	−	−
31	Kumble et al. (2022)	M	2y	c.1884C > G, p.(Phe628Leu)	Missense	Normal	−	−	−	−	+	−	−	−
32	Kumble et al. (2022)	F	11y	c.1896-2A > G, p.(?)	Splicing	Mild	+	+	−	−	−	−	−	+
33	Ververi et al. (2018)	F	14y	c.1953dup, p.(Arg652Alafs*9)	Frameshift	Moderate	+	+	−	−	−	−	−	−
34	Kumble et al. (2022)	F	4y	c.1954C > T, p.(Arg652*)	Nonsense	Moderate	+	+	−	−	+	+, 18 m	−	+
35	Kumble et al. (2022)	F	4y	c.1954C > T, p.(Arg652*)	Nonsense	Mild	+	+	−	−	−	−	−	−
36	Ververi et al. (2018)	M	8y	c.1954C > T, p.(Arg652*)	Nonsense	Mild	+	−	−	−	+	−	−	−
37	Föhrenbach et al. (2021)	M	5.5y	c.1954C > T, p.(Arg652*)	Nonsense	Mild–Moderate	+	+	−	−	−	−	+	+
38	Föhrenbach et al. (2021)	F	2.75y	c.2207G > A, p.(Ser736Asn)	Missense	Moderate	+	−	−	+	−	−	−	−
39	Kumble et al. (2022)	M	21m	c.2216G > A, p.(Trp739*)	Nonsense	Mild	+	−	−	+	−	−	−	−
40	Present study	M	5m	c.1282dup, p.(Gln428Profs*27)	Frameshift	Mild–Moderate	+	−	−	+	−	+,5 m	−	+
41	Xu et al. (2023)	F	1d	c.1896-2A > G, p.(?)	Splicing	Mild–Moderate	/	+	/	/	/	/	/	+
42	Roddate et al. (2023)	F	49y	c.337C > T, p.(Gln113*)	Nonsense	Mild	+	−	−	+	−	−	−	−
43	Roddate et al. (2023)	F	17y	c.337C > T, p.(Gln113*)	Nonsense	Mild	+	−	−	+	−	+,13y	+	+
44	Akula et al. (2023)	/	/	c.304del, p.(Val102Phefs*144)	Frameshift	/	+	/	/	/	/	/	/	+
45	Akula et al. (2023)	/	/	c.1150_1153del, p.(Phe384Glnfs*5)	Frameshift	/	+	/	/	/	/	/	/	+
46	Pande et al. (2024)	F	12y	c.1585dup, p.(Cys529Leufs*13)	Frameshift	/	/	/	/	/	/	/	/	/

+, present: feature assessed and present; −, absent: feature assessed and not present; /, unavailable: no data available or not assessed; ID, intellectual disability; Pt, patient; Ref., reference; M, male; F, female; d, day; m, month; y, year.

## 3 Results

### 3.1 Clinical data of the patient

The patient was a 5-month-and-22-day-old male infant admitted to the Department of Neurology, Hunan Children’s Hospital on July 28, 2025, due to nodding attacks for 10 days. Ten days prior to admission, the patient developed nodding attacks without obvious triggers, presenting as clustered flexor spasms with embracing arm posture. Each cluster consisted of more than 10 consecutive spasms, occurring 5–6 times per day, sometimes accompanied by irritability and crying. No fever, cough, vomiting, or diarrhea was observed.

Personal history: G_2_P_2_, delivered by full-term cesarean section with a birth weight of 2.7 kg. Routine postnatal examinations revealed short stature (−3SD < body length < −2SD), mild underweight (−2SD < body weight < −1SD), and mild microcephaly (−2SD < head circumference < −1SD). Developmental status: Global developmental delay was present before seizure onset. No developmental regression or plateau was observed prior to the onset of spasms.

Physical examination on admission: Body weight 8 kg (current good nutritional status, previous malnutrition), body length 63.0 cm, head circumference 41 cm. The patient was conscious but showed poor response. Dysmorphic features included broad forehead, depressed nasal bridge, small chin, and broad bulbous nasal tip. Two hypopigmented macules (0.3 × 0.3 cm) were noted on the abdomen. Pupils were equal and reactive to light. Cardiopulmonary and abdominal examinations were unremarkable. The patient moved all limbs freely with normal muscle tone, preserved knee tendon reflexes, and bilaterally negative Babinski sign.

**Figure 1:**
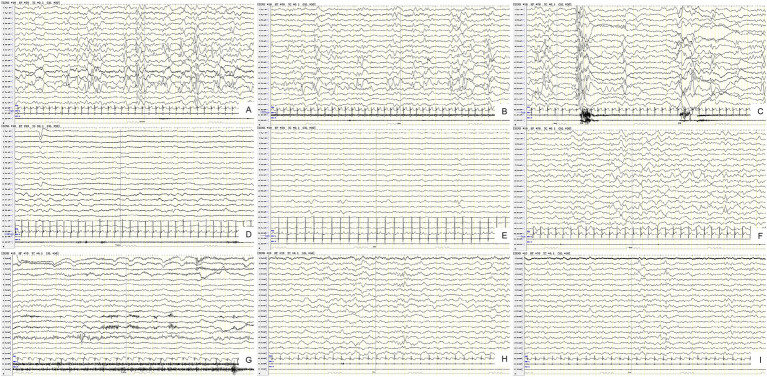
Electroencephalogram (EEG) findings of the patient with Ververi–Brady syndrome caused by *QRICH1* gene variant. **(A–C)** Baseline EEG before treatment. **(A)** (Waking): Interictal—The background was mildly slow. **(B)** (Sleep): Interictal—Abundant moderate-to-very high amplitude spikes, sharp waves, and 1.5–3 Hz sharp-slow wave discharges, predominantly in parietal, occipital, and mid-posterior temporal regions (atypical hypsarrhythmia); **(C)** (Sleep): Ictal—Electrographic correlate of clustered spasms: generalized high-amplitude slow wave with superimposed fast activity and synchronous bilateral deltoid EMG bursts. **(D–F)** Follow-up EEG before discharge (28 days after treatment initiation, 13 days after seizure cessation). **(D)** (Waking): Interictal—Mildly slowed background with increased delta waves in the temporal regions; **(E,F)** (Sleep): Interictal—Mild sharp-slow wave discharges in the temporal regions. **(G–I)** Follow-up EEG at 6 months after discharge (seizure-free for >6 months). **(G)** (Waking): Interictal—Moderately increased delta activity predominantly in the temporal lobes; **(H,I)** (Sleep): Interictal—Occasional sharp waves and sharp-slow waves mainly in the frontal and central regions.

**Figure 2:**
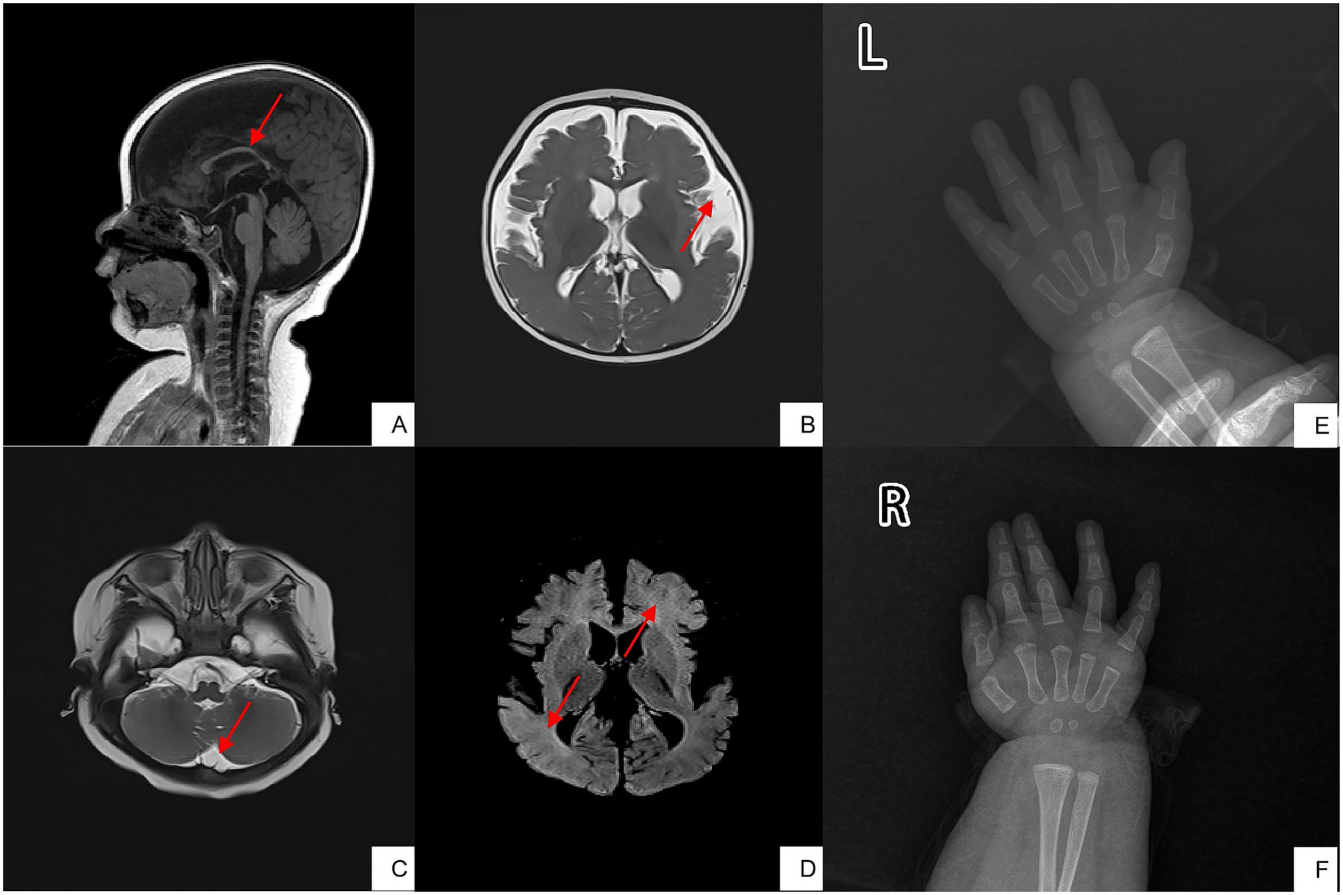
Imaging findings of the patient with Ververi–Brady syndrome caused by *QRICH1* gene variant. **(A–D)** Brain magnetic resonance imaging (MRI). **(A)** Sagittal T1WI showing thin corpus callosum; **(B)** Axial T2WI showing widened frontotemporal extra-axial spaces and dilated lateral ventricles; **(C)** Axial T2WI showing enlarged cisterna magna; **(D)** Axial FLAIR showing diffusely increased signal intensity in bilateral cerebral white matter. **(E,F)** X-rays of both hands showing no obvious bony abnormalities.

Auxiliary examinations: Routine blood, urine, and stool tests were normal. Electrolytes, liver and renal function, myocardial enzymes, and blood lactate were within normal limits. Tandem mass spectrometry of blood and urine, thyroid function, auditory brainstem response, and fundoscopy were all normal. Echocardiography showed mild tricuspid regurgitation. The Gesell Developmental Schedule revealed a score of 69 for adaptive behavior (mild developmental delay), 66 for gross motor (mild delay), 38 for fine motor (mild delay), 48 for language (moderate delay), and 47 for personal-social skills (moderate delay). EEG: Background (waking): background was mildly slow. Interictal (waking and sleep): Abundant moderate-to-very high amplitude spikes, sharp waves, and 1.5–3 Hz sharp-slow wave discharges, predominantly in parietal, occipital, and mid-posterior temporal regions, consistent with atypical hypsarrhythmia. Ictal: Clustered epileptic spasms during wakefulness (≈20 per cluster) and occasionally during sleep. Each spasm was associated with a generalized high-amplitude slow wave with superimposed low-amplitude fast activity, lasting 1–1.5 s, accompanied by synchronous bilateral deltoid EMG bursts (Figure 1). X-ray of both hands: No obvious bony abnormalities were found in the carpal, metacarpal, and phalangeal bones. Brain magnetic resonance imaging (MRI): Diffusely increased T2WI signal intensity in the cerebral white matter of both hemispheres; widened extra-axial spaces in the bilateral frontotemporal regions; enlarged cisterna magna; dilated and deformed bilateral lateral ventricles; and a thin corpus callosum (Figure 2).

### 3.2 Genetic testing results

**Figure 3:**
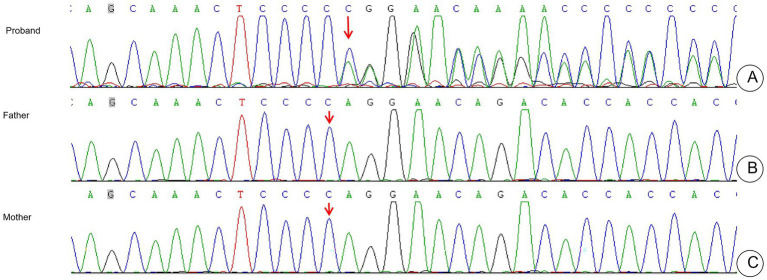
Sanger sequencing traces of the *QRICH1* gene in the proband, father, and mother. The patient carried a heterozygous frameshift variant NM_198880.3: c.1282dup (p. Gln428Profs*27). The same variant was not detected in either parent.

A novel heterozygous frameshift variant was identified in *QRICH1* by trio-WES and confirmed by Sanger sequencing: NM_198880.3: c.1282dup, p. Gln428Profs*27. The variant was absent in both parents, confirming a *de novo* origin. This variant was a duplication at nucleotide position 1,282 in the coding region, causing a frameshift starting at amino acid 428 and introducing a premature termination codon after 26 altered amino acids. According to the ACMG guidelines, ClinGen, and the expert consensus on clinical genetic testing in China, this variant was classified as pathogenic (PVS1 + PS2 + PM2_Supporting). The detailed evidence is as follows: 1. This frameshift variant occurs in an exon of transcript NM_198880.3 (total 11 exons), leading to an out-of-frame transcript and premature stop codon, which likely causes protein truncation or nonsense-mediated mRNA decay, resulting in loss of function (LoF) of the *QRICH1* protein. Truncating variants downstream of this site have been confirmed as pathogenic (PVS1). 2. *De novo* status was confirmed by trio-WES with genetically confirmed parentage and subsequently verified by Sanger sequencing (Figure 3) (PS2). 3. The variant is not recorded in the gnomAD population database (PM2_Supporting). 4. The variant has not been reported in the HGMD or ClinVar databases.

In addition to the causal *QRICH1* variant, trio-WES identified one pathogenic variant in *CLPB* and three variants of uncertain significance (VUSs) in *CLCN2*, *DYNC1H1*, and *RIC1* (Table 2). The *CLPB* variant is associated with autosomal recessive disorders and cannot explain the proband’s phenotype in heterozygous status. The three VUSs are maternally inherited and show no clinical relevance to the patient’s neurodevelopmental disorder or IESS. No other pathogenic or likely pathogenic variants in genes related to developmental and epileptic encephalopathies were detected. All alternative molecular etiologies and dual diagnosis were therefore sufficiently excluded. The VUSs will be continuously monitored for potential reclassification in future database updates.

**Table 2:** Summary of additional variants identified by trio-WES and exclusion of alternative etiologies.

Gene	Transcript	Variant	Zygosity	ACMG classification	Inheritance	Clinical relevance to the proband’s phenotype	Conclusion
*CLPB*	NM_030813.6	c.266del, p. Gly89Aspfs*152	Heterozygous	Pathogenic	De novo	Associated with autosomal recessive disorders; inconsistent with the patient’s phenotype	Non-causal
*CLCN2*	NM_004366.6	c.1310 T > C, p. Ile437Thr	Heterozygous	VUS	Maternal	Maternally inherited; mother has no related phenotype	Non-relevant
*DYNC1H1*	NM_001376.5	c.5896C > T, p. Arg1966Trp	Heterozygous	VUS	Maternal	Inconsistent with the patient’s neurodevelopmental phenotype	Non-relevant
*RIC1*	NM_020829.4	c.2393A > G, p. Tyr798Cys	Heterozygous	VUS	Maternal	Autosomal recessive disease; heterozygous without second hit	Non-relevant
*QRICH1*	NM_198880.3	c.1282dup, p. Gln428Profs*27	Heterozygous	Pathogenic	De novo	Fully consistent with Ververi–Brady syndrome and IESS	Causal variant

No other pathogenic/likely pathogenic or clinically relevant VUSs in epilepsy/neurodevelopmental disorder genes were detected. Alternative molecular etiologies were completely excluded.

### 3.3 Treatment and follow-up results

During hospitalization, the patient was administered vigabatrin at an initial dose of 125 mg q12h, which was gradually titrated to 375 mg every morning and 500 mg every night. Concurrently, ACTH was given at 20 IU for 14 days followed by 30 IU for another 14 days. Nodding spasms ceased on the second day after increasing ACTH to 30 IU. Follow-up EEG showed significant improvement: (1) abundant delta activity in the temporal regions, predominantly on the left side; (2) frequent sharp waves and sharp-slow wave discharges over bilateral temporal regions, left greater than right (Figure 1).

At an outpatient follow-up of more than 6 months, no spasms recurred. Repeated EEG revealed mild delta activity mainly in the temporal lobes, and occasional sharp waves and sharp-slow wave discharges over the frontal and central regions (Figure 1). The background EEG activity improved and epileptiform discharges were further reduced. Follow-up Gesell Developmental Schedule scores were as follows: adaptive behavior 69, gross motor 67, fine motor 67, language 79, personal-social 71, indicating improved developmental delay.

### 3.4 Literature review results

Using the predefined search strategy, 11 eligible studies were retrieved (Kumble et al., 2022; Ververi et al., 2018; Baruch et al., 2021; Föhrenbach et al., 2021; Cope et al., 2020; Lui et al., 2019; Wang and Wu, 2023; Xu et al., 2023; Roddate et al., 2023; Akula et al., 2023; Pande et al., 2024). Including the present case, there were 46 patients with *QRICH1* variants reported worldwide. Detailed clinical data and variant loci are summarized in Table 1. A total of 41 distinct *QRICH1* variants were identified, including 10 missense variants and 31 loss-of-function (LoF) variants (11 nonsense, 17 frameshift, and 3 splicing variants). Among the 46 patients, clinical information was incomplete or limited for some individuals. Phenotype frequencies were calculated separately for each feature using only patients with available data for that phenotype. Sex distribution: 22 males, 22 females; sex was unavailable for 2 patients. Age at first clinical evaluation ranged from the neonatal period to 49 years. The main clinical features included mild to severe developmental delay/intellectual disability (30/41, 73.2%), non-specific facial dysmorphism (41/44, 93.2%), hypotonia (15/43, 34.9%), poor weight gain (12/42, 28.6%), short stature (16/42, 38.1%), autism spectrum disorder (11/42, 26.2%), epilepsy (11/42, 26.2%), and scoliosis (8/42, 19.0%). Structural brain abnormalities were reported in 33.3% (15/45) of patients who underwent brain imaging. Age at seizure onset ranged from 5 months to 14 years; our patient had the earliest reported seizure onset. Various seizure types were observed, including infantile spasms, absence seizures, atonic seizures, and focal seizures, all accompanied by generalized EEG abnormalities.

## 4 Discussion

The *QRICH1* gene is associated with autosomal dominant neurodevelopmental disorder Ververi–Brady syndrome (VBS), a rare condition first reported in 2018 (Ververi et al., 2018). Clinical features of this syndrome include developmental delay, intellectual disability, mild facial dysmorphism, hypotonia, short stature, autism spectrum disorder, and epilepsy. Additional variable features include scoliosis, cardiac, renal, and brain structural anomalies (Kumble et al., 2022). To date, most variants identified in *QRICH1* are nonsense and frameshift variants, thought to act through loss-of-function (LoF) mechanisms. A small number of patients carry missense variants, who also present with speech and motor delay as well as cognitive impairment. In the present study, we identified a novel *de novo* frameshift variant c.1282dup (p. Gln428Profs*27), which has not been previously reported, thereby expanding the variant spectrum of this gene. The patient mainly presented with mild facial dysmorphism, short stature, global developmental delay, and epilepsy.

A recent study demonstrated that *QRICH1* acts as a transcriptional regulator involved in endoplasmic reticulum (ER) homeostasis by modulating protein translation and secretion (You et al., 2021; Wang et al., 2025; Bianchi et al., 2025). Variants in *QRICH1* may lead to neurodevelopmental disorders through dysregulated ER stress responses, as impaired secretory pathways have been shown to compromise synapse formation and/or function during neural development (Martínez et al., 2018; Mignogna et al., 2015). The novel heterozygous frameshift variant identified in this study (c.1282dup, p. Gln428Profs*27) is predicted to induce nonsense-mediated mRNA decay and lead to complete LoF of QRICH1. Such loss of function may impair synapse formation and function, interfere with postsynaptic signal transmission, and further disrupt normal neuronal development and function. As one of the main clinical manifestations, epilepsy may be closely associated with synaptic dysfunction caused by *QRICH1* variants. Epileptogenesis may be attributed to imbalanced excitability of neural networks resulting from abnormal synaptic transmission (Davletshin et al., 2023). Previous studies have reported that variants in synaptic-related genes frequently lead to developmental and epileptic encephalopathies (Guerrini et al., 2023). Therefore, *QRICH1* variants may trigger seizures through similar pathways. Further studies should focus on the specific role of *QRICH1* protein in synaptic signaling and its interaction with other synaptic proteins to elucidate the underlying molecular pathogenic mechanism.

Based on the clinical and genetic data of 46 patients summarized in Table 1, a tentative genotype–phenotype correlation can be observed in *QRICH1*-related Ververi–Brady syndrome. LoF variants, including frameshift, nonsense, and splicing variants, accounted for the majority of pathogenic alleles and were associated with a broad spectrum of severity ranging from mild to severe intellectual disability, developmental delay, early-onset seizures, and brain malformations. In contrast, missense variants were predominantly associated with milder or moderate phenotypes, including milder intellectual disability and a lower frequency of severe epilepsy or structural brain anomalies. Our patient harbored a *de novo* frameshift variant (c.1282dup, p. Gln428Profs*27), a typical LoF allele, and presented with mild-to-moderate developmental delay, short stature, and infantile epileptic spasms syndrome starting at 5 months of age. This clinical presentation is consistent with the relatively broader and more severe phenotypic spectrum observed among individuals with LoF variants.

To date, only 10 patients with *QRICH1*-related disorders have been reported to have epilepsy. The age at seizure onset among these 10 patients ranged from 6 months to 14 years. Seizure types were diverse, including infantile spasms, absence seizures, atonic seizures, focal seizures, and bilateral tonic–clonic seizures, all accompanied by generalized EEG abnormalities (Kumble et al., 2022). Among these 10 patients, only one case described antiepileptic treatment and outcome. That patient developed focal motor seizures of the hand at age 13 years, progressing to bilateral tonic–clonic seizures. Treatment with oxcarbazepine and levetiracetam was ineffective, with worsening EEG and evolution into epileptic encephalopathy (Roddate et al., 2023). The patient reported here developed seizures at 5 months of age, representing the earliest reported seizure onset in patients with *QRICH1*-related disorders. The patient mainly presented with clustered spasms, which were well controlled after treatment with ACTH and vigabatrin, with resolution of hypsarrhythmia on EEG and improvement in developmental delay. In this patient, combined treatment with ACTH and vigabatrin resulted in clear clinical and electrographic improvement, with sustained seizure freedom and developmental gains. This report presents descriptive clinical experience regarding IESS management in this individual patient with a *QRICH1*-related disorder. Vigabatrin increases brain levels of the inhibitory neurotransmitter GABA through irreversible inhibition of GABA transaminase, thereby reducing the excitability of neural networks (Kuchenbuch et al., 2024). As an adrenocorticotropic hormone, ACTH has been widely used for the treatment of infantile spasms, but its precise mechanism in *QRICH1*-related epilepsy remains to be clarified. We hypothesize that ACTH may exert antiepileptic effects by regulating immune dysregulation (Wanigasinghe et al., 2021). Previous studies have shown that *QRICH1* mediates CD8 + T-cell activation via CARD11, and *QRICH1* deficiency broadly affects the expression of immune-related genes, leading to immune dysregulation (Carter et al., 2025). Immune dysregulation may contribute to the formation of an inflammatory microenvironment in the brain, exacerbating neuronal injury and seizures. As an immunomodulator, ACTH can improve the cerebral immune microenvironment, alleviate neuronal damage, and thereby control seizures by suppressing excessive inflammatory responses and regulating immune cell functions (Kaczorowska et al., 2023).

Short stature, chondrodysplasia, scoliosis, and delayed bone age indicate that *QRICH1* protein plays a role in regulating skeletal growth and development. Lui et al. identified chondrodysplasia in two previous individuals and demonstrated that reduced QRICH1 expression in a mouse model leads to downregulation of several genes involved in chondrocyte hypertrophic differentiation, resulting in impaired longitudinal bone growth (Lui et al., 2019). Kumble et al. reported that 18% of examined individuals had chondrodysplasia (Kumble et al., 2022). The patient reported here had short stature, but hand X-ray at 6 months of age showed no obvious abnormalities. Chondrodysplasia may be an age-dependent feature that varies with skeletal maturation. Therefore, further radiological follow-up is required to determine the specificity of chondrodysplasia in this disorder. Kumble et al. showed that two out of three patients treated with growth hormone responded to this intervention, suggesting that growth hormone therapy may ameliorate the skeletal effects of complete or partial *QRICH1* deficiency (Kumble et al., 2022). Thus, regular assessment of height and chondrodevelopment in this patient during follow-up is necessary for early detection and active management.

Kumble et al. performed brain imaging in 19 individuals, and 53% showed mild abnormalities including non-specific periventricular white matter anomalies, arachnoid cysts, and pineal cysts (Kumble et al., 2022). In addition, Akula et al. identified two patients with rare parietal-predominant polymicrogyria, reporting this finding for the first time as a phenotype associated with *QRICH1* variants (Akula et al., 2023). Only one Chinese paper on *QRICH1* variants has been published, reporting a patient with bilateral ventricular enlargement on prenatal ultrasound, who underwent ventriculoperitoneal shunt at 1 month and 25 days of age due to progressive ventricular dilatation (Xu et al., 2023). Roddate et al. reported a female patient with leukodystrophy on brain MRI (Roddate et al., 2023). The present patient also showed abnormal cerebral white matter signals in both hemispheres on brain MRI; this imaging phenotype has been rarely reported in *QRICH1*-related VBS, and its clinical significance warrants further investigation. Abnormal white matter signals may serve as an important auxiliary imaging diagnostic clue for *QRICH1*-related VBS. Furthermore, the extent and severity of white matter abnormalities may reflect the degree of neurological injury. The diffuse mildly increased white matter signal in this patient, accompanied by developmental delay, suggests a potential correlation. In long-term follow-up, repeated MRI can dynamically monitor changes in white matter signals, evaluate disease progression and treatment efficacy, and provide evidence for clinical diagnosis and management. However, care should be taken to differentiate this condition from other neurodevelopmental disorders such as hereditary myelin diseases and metabolic encephalopathies to avoid misdiagnosis.

In addition to the above phenotypic features, other patients have been reported to have variable hypotonia, elevated myocardial enzymes, autism spectrum disorder, feeding difficulties, gastroesophageal reflux, cardiac anomalies, renal anomalies, microcephaly, etc. (Baruch et al., 2021; Wang and Wu, 2023; Pande et al., 2024). Föhrenbach reported one patient with Wilms tumor. To our knowledge, no other reports have linked *QRICH1* to Wilms tumor or other neoplasms (Föhrenbach et al., 2021). No other reported patients with *QRICH1* variants have developed Wilms tumor or childhood cancers. No such abnormalities were found in our patient, and further follow-up is needed.

In summary, this single-case study enhances understanding of VBS caused by *QRICH1* variants and documents the observed clinical, electrographic, and developmental course following treatment with ACTH combined with vigabatrin in this patient. It also provides supportive evidence for the recognition and individualized clinical care of such rare genetic neurodevelopmental disorders.

Limitations: First, the findings are based on a single case report, which may restrict the generalizability of the treatment response and genotype–phenotype correlation observed. Second, the clinical follow-up duration is limited, preventing definitive conclusions regarding long-term neurological and developmental outcomes. Third, trio-based whole-exome sequencing only detects SNVs and small indels, and cannot identify CNVs, structural variations, or repeat expansions; however, no clinical or auxiliary evidence supported other etiologies. Fourth, no functional experiments were performed to verify the pathogenicity of the novel *QRICH1* variant. Finally, the literature review is descriptive, and incomplete records in published cases may limit the accuracy of genotype–phenotype analyses. Larger cohorts, longer follow-up, and functional studies are needed in future research.

## Data Availability

The data presented in the study are deposited in the ClinVar repository (https://www.ncbi.nlm.nih.gov/clinvar/), accession number SUB16244878.
